# High lipid, low dextrose parenteral nutrition allows patient to achieve nutritional autonomy: A case report

**DOI:** 10.1016/j.ijscr.2023.108510

**Published:** 2023-07-19

**Authors:** Andrew Adorno, Jennifer Silinsky, Michael Ghio, Nathaniel Rogers, John Tyler Simpson, Chrissy Guidry

**Affiliations:** Department of Surgery, Tulane Medical Center, New Orleans, LA 70112, USA

**Keywords:** Parenteral nutrition, Lipid, Fish oil, Dextrose, Liver disease, Case report

## Abstract

**Introduction:**

Prolonged use of parenteral nutrition can eventually lead to liver abnormalities. Causative factors include decreased enteral stimulation, high intakes of intravenous dextrose, proinflammatory 100 % soybean oil-based lipids, and increased burden on liver through 24-h infusions. We present a case report of a patient who received parenteral nutrition modifications to address liver dysfunction.

**Presentation of case:**

Our patient was a 37-year-old African American male with a past medical history including refractory Crohn's disease complicated by multiple small bowel obstructions, several bowel surgeries, left lower quadrant colostomy placement, short bowel syndrome, severe protein calorie malnutrition, parenteral nutrition dependence, and elevated liver function tests. He was admitted for nutritional optimization before a planned takedown of multiple chronic enterocutaneous and perianal fistulas. His home parenteral nutrition order contained high amounts of dextrose (69 % kcal), and low amounts of 100 % soybean oil (11 % kcal).

**Discussion:**

Due to an elevated alkaline phosphatase level at baseline (1746 U/L), the Registered Dietitian maximized protein, decreased the dextrose by 62.5 %, and changed to SMOFlipid (a fish-oil containing lipid) at >1 g/kg/day to address liver abnormalities. Within 1.5 months of changing parenteral nutrition to high SMOFlipid (>30 % kcal) with low dextrose (<30 % kcal) content, alkaline phosphatase levels declined by 62 %, prealbumin levels increased by 56 %, and c-reactive protein levels decreased by 62 %.

**Conclusion:**

Parenteral nutrition modifications led to reversal of chronic liver dysfunction. This patient ultimately underwent a successful high-risk fistula takedown procedure, allowing for complete weaning of parenteral nutrition and achievement of sustained nutritional autonomy.

## Highlights

• Higher amounts of SMOFlipid and less dextrose should be considered in parenteral nutrition.
• This new dosing approach can allow patients to meet higher calorie targets with less risk.
• Dosing SMOFlipid >1 g/kg and/or > 30 % of kcal and dextrose <30 % of kcal is safe.
• Chronic liver abnormalities can improve within a relatively short period with this approach.

## 1 Introduction

Intestinal failure associated liver disease (IFALD) is a multifactorial condition characterized by cholestasis, hepatic steatosis, and fibrosis related to parenteral nutrition (PN) use [1]. The longer a patient receives PN, the more likely they are to develop IFALD [2]. IFALD may have a variety of causative factors including decreased enteral stimulation (can lead to bile acid accumulation in the liver), 100 % soybean oil injectable lipid emulsion (SO-ILE) (contain pro-inflammatory omega-6 long chain fatty acids and phytosterols), high amounts of dextrose (can increase insulin secretion and stimulate lipogenesis leading to hepatic fat accumulation), and 24-h PN infusions (can be an increased burden on the liver via constant processing of intravenous calories) [1,3,4].

Nutrition interventions are typically the first line therapy to address IFALD (e.g., reducing the infusion time of PN, reducing calorie targets, decreasing dextrose content, and limiting SO-ILE to reduce the infusion's burden on the liver) [1,3,4]. High amounts of calories from dextrose specifically have been linked to liver abnormalities in long term home PN patients [2,5,6]. To minimize risk of dextrose and SO-ILE via PN, recommendations include prescribing dextrose <65 % total kcal, limiting dextrose to <4 g/kg/day, maintaining a glucose infusion rate of <4 mg/kg/min, and dosing SO-ILE <1 g/kg/day [1,2,4,7]. However, utilizing a combination of these nutrition strategies increases the risk of underfeeding. Newer generation lipids containing fish oil can assist with optimizing non-dextrose calories in PN and ameliorate the pro-inflammatory effects of SO-ILE [8]. SMOFlipid® (Fresenius Kabi) is a mixed lipid emulsion containing 30 % soybean oil, 30 % medium-chain triglycerides, 25 % olive oil, and 15 % fish oil [9]. We present a retrospective case report of a patient who received higher doses of SMOFlipid to address IFALD which resulted in successful surgical intervention, complete weaning of PN, and achievement of nutritional autonomy. This patient was managed at an acute care, inpatient, academic, tertiary referral hospital in the United States. This case was reported in line with SCARE criteria [10].

## 2 Case presentation

The patient discussed in this report is a 37-year-old African American male with a body mass index (BMI) of 18.6 and a past medical history including refractory Crohn's disease complicated by multiple small bowel obstructions, several bowel surgeries, left lower quadrant colostomy placement, short bowel syndrome (SBS), severe protein calorie malnutrition, PN dependence, and elevated liver function tests (LFTs). He is an ambulatory non-smoker without alcohol or illicit drug use and only has a family history of paternal hypertension. He initiated PN in May 2019, began to have elevated alkaline phosphatase (ALP) levels >200 U/L in July 2019, and sustained ALP levels >1000 U/L since June 2020. He presented to our facility in October 2021 for nutritional optimization before a planned takedown of chronic lower abdominal enterocutaneous fistulas (x3) and a perianal fistula.

His baseline ALP level upon admission was 1746 U/L. This patient also had significant challenges maintaining central venous access, as his only access site was a femoral line. Prior to admission, pre-intervention measures taken to reduce the inflammatory burden on the liver was to decrease the dose of SO-ILE and condense the PN infusion time to 12 h. However, due to elevated nutrition needs for multiple fistulas and severe malnutrition, more calories from dextrose were provided to compensate for reduction of SO-ILE, and the infusion was transitioned back to 24 h to replace fistula losses. His home PN order provided an average of 33.5 kcal/kg with 69 % of calories from dextrose and only 11 % of calories from SO-ILE (Table 1).

**Table 1:** Parenteral Nutrition Composition.

Dosing Weight: 58.9 kg, BMI 18.6		
Nutrient	Prior to admission	During admission
Calories (kcal)	1974	1750
kcal/kg/day	33.5	30
Amino Acids (g)	100	160
% kcal (%)	20	37
g/kg/day	1.7	2.7
Dextrose (g)	400	150
% kcal (%)	69	29
g/kg/day	6.8	2.5
GIR (mg/kg/min)	4.7 (24 h), 9.4 (12h)	1.8 (24 h), 3.5 (12 h)
Lipid (g)	SO-ILE	SMOFlipid
Frequency	Three Times Weekly	Daily
Amount g/week	150	420
Average g/day	21.4	60
% kcal (%)	11	34
g/kg/day	0.36	1.02

kcal, kilocalories; kg, kilograms; g, grams; %, percent; GIR, glucose infusion rate; SO-ILE, 100 % soybean oil intravenous lipid emulsion; BMI, body mass index.

There was no delay from presentation to intervention, and the primary type of intervention strategy was nutritional via modification of PN composition. Other peri-interventional strategies included vitamin D supplementation for a baseline deficiency (12.8 ng/dL) and initiation of 3 weeks of ursodeoxycholic acid by the hepatology team. Pertinent medication use throughout admission is outlined for review (Table 2).

**Table 2:** Medications throughout admission.

Medication	Dose	Date	Number of Doses
Ergocalciferol	50,000 IU Weekly	11/17–12/8	4 doses
Cholecalciferol	1000 IU Daily	11/6–11/17	
Hydromorphone	2 mg IV q 4 h	During Admit	
Ferrous sulfate	325 mg PO	During Admit	
Piperacillin / Tazobactam	3.375 g q 8 h	11/23–12/7	
Neomycin	1000 mg × 3 doses		1 day
Micafungin	100 mg IV q 24 h	11/23–11/29	
Daptomycin	500 mg IV q 24 h	11/23–11/29	
Ursodeoxycholic acid	300 mg PO BID	11/15–12/8	
Prednisone	60 mg BID	12/1–12/2	2 doses
	40 mg BID	12/2–12/3	2 doses
Methylprednisolone	16 mg BID IV	12/3–12/4	2 doses
	8 mg BID IV	12/4–12/5	2 doses
	4 mg BID IV	12/5–12/6	2 doses
	32 mg IV	12/10	1 dose
	16 mg IV	12/11	1 dose
	8 mg IV	12/12	1 dose

IU, International Unit; IV, Intravenous; PO, by mouth; BID, twice daily.

Upon admission to our facility for nutrition optimization, the Registered Dietitian increased the protein to the maximum level tolerated (2.7 g/kg, 37 % kcal), decreased the dextrose by 62.5 %, and changed the SO-ILE to SMOFlipid with a goal of >1 g/kg/day as a total nutrient admixture (amino acids, dextrose, and lipids in the same bag) (Table 1). For the first 22 days, TPN was provided as a 24-h infusion to replenish fistula losses. However, on day 23 the infusion was transitioned to cyclic over 12 h to allow for liver rest. To ensure consistency in administration, the patient received PN and lipids daily throughout admission.

**Fig. 1:**
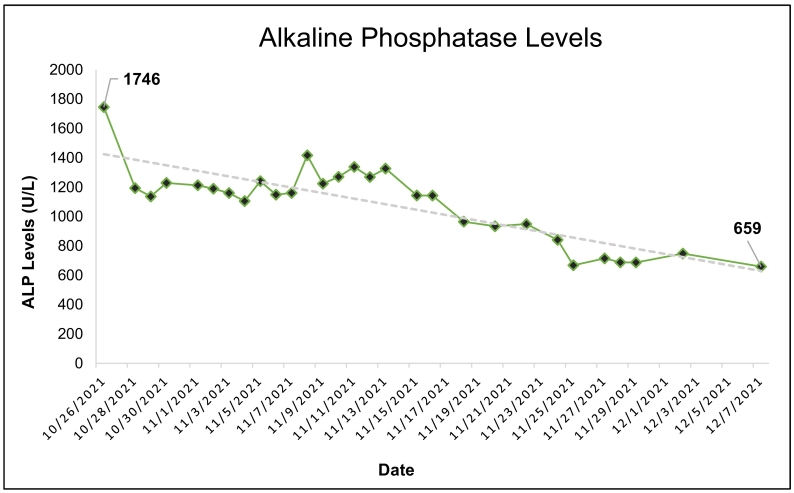
Progression of Alkaline Phosphatase Levels Downward trend of alkaline phosphatase (ALP) levels from 1746 U/L to 659 U/L during 1.5-month inpatient hospitalization prior to surgical intervention.

**Fig. 2:**
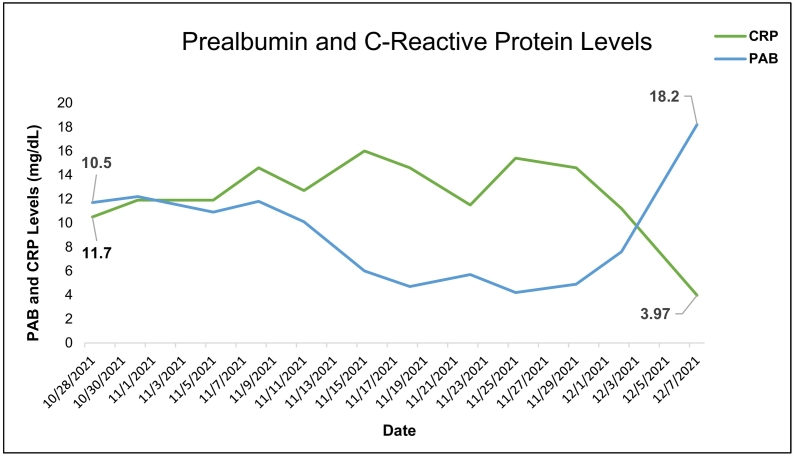
Progression of Prealbumin and C-Reactive Protein Levels Inverse progression of prealbumin (PAB) and c-reactive protein (CRP) levels as liver function simultaneously improved.

**Fig. 3:**
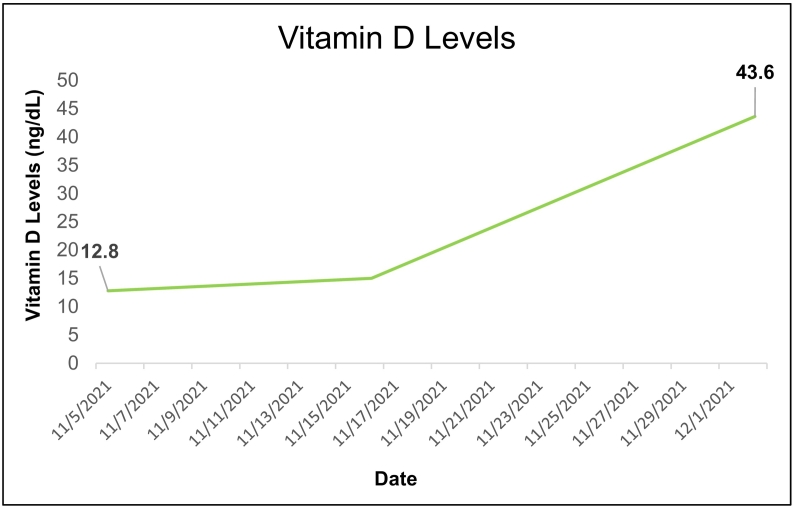
Progression of Vitamin D Levels Correction of baseline vitamin D deficiency during admission via 25(OH)D testing.

Over 44 days (about 1.5 months), ALP levels decreased by about 62 % from 1746 U/L to 659 U/L (Fig. 1). Prealbumin (PAB) levels increased by about 56 %, and c-reactive protein (CRP) levels decreased by about 62 % (Fig. 2). Vitamin D levels also increased by about 71 % (Fig. 3). There were no complications or adverse/unanticipated events, and the patient tolerated treatment without laboratory abnormalities. Serum triglyceride levels peaked at 115, renal function remained within normal limits, normoglycemia was maintained throughout admission with no episodes of hyperglycemia (>180 mg/dL) or hypoglycemia (<70 mg/dL), and body weight remained stable.

## 3 Discussion

A variety of management approaches are typically needed to address the liver dysfunction associated with IFALD. Medically, the patient can be given hepatoprotective agents (e.g., ursodeoxycholic acid) to improve bile flow/inflammation and antibiotics (e.g., metronidazole) to assist with intestinal overgrowth of anaerobic bacteria that can lead to elevated LFTs [11,12]. Nutritionally, fish oil-based lipid emulsions containing omega-3 fatty acids may assist with improving LFTs by reducing liver inflammation, improving insulin resistance, and decreasing lipid accumulation in the liver [1,8,[13], [14], [15]]. There are other completely plant-based mixed oils available to reduce omega-6 fatty acid and phytosterol intake, however SMOFlipid has the lowest phytosterol content and the highest amount of omega-3 fatty acids comparatively [8].

Serum PAB was used as a surgical marker to evaluate the likelihood of improved surgical outcomes in this high-risk SBS patient [16]. PAB is primarily synthesized in the liver and is a negative acute-phase reactant (tends to decrease with inflammation), while CRP levels tend to increase with inflammation [16]. Therefore, a variety of nutritional strategies were utilized to focus on correction of LFTs and decreasing inflammatory components of PN (e.g., decreasing dextrose, increasing protein, addition of fish oil, encouragement of oral intake despite malabsorptive component of SBS, cycling the PN) in order to indirectly optimize PAB and CRP levels to prepare for surgery.

Our analysis has both strengths and limitations. The primary strength of this descriptive analysis is the time in which reversal of 2.25 years of IFALD began to occur using SMOFlipid (44 days). Using higher doses of SMOFlipid allowed us to meet our patient's elevated calorie needs (30 kcal/kg) while simultaneously addressing liver abnormalities. Although all potential LFT confounders were unable to be completely mitigated (e.g., oral intake, cyclic PN, antibiotics, gallstone dissolvents), we feel the primary intervention was modification of PN lipid and dextrose content. Our patient was on a solid oral diet prior to admission, so the degree of enteral stimulation was not different at baseline. Furthermore, cyclic PN prior to admission did not lead to improvements in LFTs. Moreover, if our patient's home PN order was made cyclic over 12 h to allow for liver rest, the glucose infusion rate would have exceeded 9.4 mg/kg/min, which is not recommended [2,7]. Additionally, metronidazole (the antibiotic provided to assist with IFALD) was not specifically prescribed [12].

Dosing guidelines for SMOFlipid are 1–2 g/kg/day [9], so applying these dosing guidelines are within manufacturer's recommendations and unlikely to pose risk if applied to a larger population. The novelty of our technique was utilizing higher doses of SMOFlipid (>1 g/kg/day, >30 % kcal) and protein (>35 % kcal) to achieve a lower dextrose solution (<30 %). A learning curve may occur for clinicians when dosing mixed lipids >1 g/kg since SO-ILE has been historically dosed minimally to prevent complications. Regarding cost, a recent international analysis showed sizable financial savings associated with fish oil containing lipids [17]. The key takeaway of our analysis shows that using higher doses of SMOFlipid and protein to minimize the complications of overfeeding dextrose can help patients prevent/treat liver abnormalities while also achieving calorie targets.

## 4 Conclusion

Prior to presentation, our patient had a 2.25-year history of liver abnormalities related to PN. Within 1.5 months of PN formulation changes, ALP levels declined by 62 %, PAB levels increased by 56 %, and CRP levels decreased by 62 %, allowing this patient to undergo a high-risk fistula takedown procedure successfully. With much gratitude our patient discontinued long term PN dependence and transitioned to meeting his nutrition needs via oral diet alone, which has continued to the present day at multiple follow up clinic appointments (e.g., 6-month, 12-month, 18-month).

In combination with classic recommendations to address liver dysfunction in patients on PN, newer generation lipids containing fish oil (specifically SMOFlipid) may allow patients to achieve higher calorie targets while preventing/treating liver abnormalities. Using higher doses of SMOFlipid (>1 g/kg, >30 % kcal) and protein in PN to provide lower amounts of dextrose (<30 % kcal) is an area that warrants exploration in future randomized control trials.

## CRediT authorship contribution statement

Writing the paper, Study Concept: Andrew Adorno, Jennifer Silinsky, Michael Ghio, Nathaniel Rogers, John Tyler Simpson, Chrissy Guidry.

Data collection, Study Concept: Andrew Adorno, Michael Ghio, Nathaniel Rogers, John Tyler Simpson.

Supervision: Jennifer Silinsky, Chrissy Guidry.

## Funding

This research did not receive any specific grant from funding agencies in the public, commercial, or not-for-profit sectors.

## Ethical approval

Ethical approval for this study (2021–181) was provided by an Institutional and Ethics Review Board in New Orleans, Louisiana, United States on 3 May 2021.

## Consent

Written informed consent was obtained from the patient for publication of this case report. A copy of the written consent is available for review by the Editor-in-Chief of this journal on request.

## Registration of research studies

N/A

## Guarantor

Andrew Adorno, Jennifer Silinsky, Michael Ghio, Nathaniel Rogers, John Tyler Simpson, Chrissy Guidry.

## Declaration of competing interest

The authors report no declarations of interest.

## References

[1] Rochling F.A. (2021). Intravenous lipid emulsions in the prevention and treatment of liver disease in intestinal failure. Nutrients..

[2] Cavicchi M., Beau P., Crenn P., Degott C., Messing B. (2000). Prevalence of liver disease and contributing factors in patients receiving home parenteral nutrition for permanent intestinal failure. Ann. Intern. Med..

[3] Howard L., Ashley C. (2003). Management of complications in patients receiving home parenteral nutrition. Gastroenterology..

[4] Klein C.J., Stanek G.S., Wiles C.E. (1998). Overfeeding macronutrients to critically ill adults: metabolic complications. J. Am. Diet. Assoc..

[5] Reimund J.M., Duclos B., Arondel Y., Baumann R. (2001). Persistent inflammation and immune activation contribute to cholestasis in patients receiving home parenteral nutrition. Nutrition..

[6] Zagara G., Locati L. (1989). Role of total parenteral nutrition in determining liver insufficiency in patients with cranial injuries. Glucose vs glucose + lipids. Minerva Anestesiol..

[7] Schloerb P.R., Henning J.F. (1998). Patterns and problems of adult total parenteral nutrition use in US academic medical centers. Arch. Surg..

[8] Leguina-Ruzzi A.A., Ortiz R. (2018). Current evidence for the use of smoflipid emulsion in critical care patients for parenteral nutrition. Crit Care Res Pract..

[9] SMOFlipid (2020).

[10] Agha RA, Franchi T, Sohrabi C, Mathew G, Kerwan A; SCARE Group. The SCARE 2020 guideline: updating consensus surgical CAse REport (SCARE) guidelines. Int. J. Surg. 2020;84:226–230. doi:10.1016/j.ijsu.2020.10.034. 33181358

[11] San Luis V.A., Btaiche I.F. (2007). Ursodiol in patients with parenteral nutrition-associated cholestasis. Ann. Pharmacother..

[12] Capron J.P., Gineston J.L., Herve M.A., Braillon A. (1983). Metronidazole in prevention of cholestasis associated with total parenteral nutrition. Lancet..

[13] Scorletti E., Byrne C.D. (2018). Omega-3 fatty acids and non-alcoholic fatty liver disease: evidence of efficacy and mechanism of action. Mol. Asp. Med..

[14] Dai Y.J., Sun L.L., Li M.Y., Ding C.L., Su Y.C., Sun L.J. (2016). Comparison of formulas based on lipid emulsions of olive oil, soybean oil, or several oils for parenteral nutrition: a systematic review and meta-analysis. Adv. Nutr..

[15] Lepretti M., Martucciello S., Burgos Aceves M.A., Putti R., Lionetti L. (2018). Omega-3 fatty acids and insulin resistance: focus on the regulation of mitochondria and endoplasmic reticulum stress. Nutrients..

[16] Sharma A., Giraddi G., Krishnan G., Shahi A.K. (2014). Efficacy of serum Prealbumin and CRP levels as monitoring tools for patients with fascial space infections of odontogenic origin: a clinicobiochemical study. J Maxillofac Oral Surg..

[17] Pradelli L., Klek S., Mayer K., Omar Alsaleh A.J., Rosenthal M.D., Heller A.R. (2021). Cost-effectiveness of parenteral nutrition containing ω-3 fatty acids in hospitalized adult patients from 5 European countries and the US. JPEN J. Parenter. Enteral Nutr..

